# A multi-antigen *Campylobacter* vaccine enhances antibody responses in layer breeders and sustains elevated maternal antibody levels in their offspring

**DOI:** 10.1016/j.psj.2025.104898

**Published:** 2025-02-17

**Authors:** Mostafa Naguib, Shreeya Sharma, Abigail Schneider, Ari. J Bragg, Khaled Abdelaziz

**Affiliations:** a Department of Animal and Veterinary Science, Clemson University, Clemson, SC 29634, USA; b Department of Poultry Diseases, Faculty of Veterinary Medicine, Cairo University, Cairo 12211, Egypt; c Clemson University School of Health Research (CUSHR), Clemson, SC 29634, USA

**Keywords:** Chicken, *Campylobacter,* CpG ODN, Maternal antibodies, Outer membrane proteins

## Abstract

*Campylobacter jejuni* is the leading cause of bacterial gastroenteritis worldwide, with an estimated 1.5 million human infections occurring annually in the United States alone. With chickens being considered the primary reservoir and source of infection in humans, developing effective vaccination strategies is crucial for preventing *Campylobacter* transmission to humans. This study aimed to examine the immunogenicity and protective efficacy of a multi-antigen subunit vaccine, consisting of *C. jejuni* outer membrane proteins (OMPs) and cytosine-phosphorothioate-guanine oligodeoxynucleotides (CpG ODN), in layer breeders and its potential to enhance the levels of *C. jejuni*-specific maternal antibodies in their offspring. Four groups of layer breeders were subcutaneously vaccinated with 200 μg *C. jejuni* OMPs and 50 μg CpG-ODN, individually or combined, or with PBS as a negative control. *C. jejuni* shedding and antibody levels were monitored in breeders for up to ten weeks post-vaccination. At the peak of antibody levels (the fourth week post-primary vaccination), fertilized eggs were collected and incubated in a sensitized egg incubator until hatching. Maternally derived antibodies (MDA) were measured in the serum of hatched chicks for five weeks post-hatch. The results revealed that breeders vaccinated with the combination of *C. jejuni* OMPs and CpG ODN exhibited a significant reduction in *C. jejuni* shedding by up to 1.37 log_10_. This group also showed significantly higher serum and egg yolk IgY and IgM levels compared to the non-vaccinated negative control group, and notably, their chicks maintained significantly higher serum IgY, IgM, and IgA levels for five weeks post-hatch compared to the negative control group. Overall, these outcomes suggest that a combination of *C. jejuni* OMPs and CpG ODN could offer a promising vaccine strategy to reduce *Campylobacter* colonization in breeders and to boost and sustain high levels of *C. jejuni*-specific MDA in hatched chicks. Further research is needed to evaluate the protective effects of vaccine-associated MDA against *Campylobacter* infection in a commercial broiler model.

## Introduction

The sustainability of the poultry sector is essential for meeting the increasing global demand for animal proteins. However, the rising incidence of foodborne illness linked to the consumption of contaminated poultry products presents a significant challenge to this sector (Sahin et al., 2015; Al Hakeem et al., 2022). Indeed, poultry vaccines are available for various foodborne bacterial pathogens, such as *Salmonella* and *Escherichia coli*, yet no commercial vaccine exists for *Campylobacter. Campylobacter* alone causes approximately 1.5 million human infections annually in the US (Scharff, 2012; Nemhauser, 2023), with poultry products being reported as the primary source of infection. Although campylobacteriosis is a typically self-limited disease, infection in immunocompromised people, the elderly and children can lead to serious health complications, such as inflammatory bowel disease, reactive arthritis, and Guillain-Barré syndrome (Epps et al., 2013). While most studies have reported that *C. jejuni* colonizes the ceca at high densities reaching up to 10^9^ colony-forming units (cfu)/gram of cecal content without causing clinical disease (Humphrey et al., 2014; Ijaz et al., 2018; Ty et al., 2022), some studies have associated its presence with poor gut health (Knudsen et al., 2006; Williams et al., 2013). Therefore, strategies to control *Campylobacter* in humans should focus on preventing clinical disease, while in chickens, the goal should be to reduce its colonization and mitigate its negative effects on the chicken gut.

*C. jejuni* is horizontally transmitted in poultry farms through environmental sources, such as contaminated diet, drinking water, or biological vectors, such as wild birds, insects and rodents (Sahin et al., 2002a; Hakeem and Lu, 2021). Once introduced into the barn by any of these sources, the entire flock becomes colonized with *Campylobacter* within a few days (Sahin et al., 2002a).

A great deal of evidence indicates that newly hatched chicks are resistant to infection with *Campylobacter* during the first two weeks of age, largely due to the relatively high levels of maternally derived antibodies (MDA) (Sahin et al., 2003; Cawthraw and Newell, 2010; Haems et al., 2024). As the chicks age, the levels of MDA gradually decline, making them more susceptible to *C. jejuni* infection. As such, enhancing and maintaining high levels of *C. jejuni*-specific MDA in hatched chicks until slaughter age could serve as a strategy to provide long-term passive protection against *Campylobacter.*

Numerous pre-harvest intervention strategies have been investigated to control *Campylobacter* colonization rates in poultry flocks (Neal-McKinney et al., 2012; Smith et al., 2016; Connerton et al., 2018; D'Angelantonio et al., 2021; Śmiałek et al., 2021), with vaccination being considered key for controlling this microorganism in chickens (Taha-Abdelaziz et al., 2023; Helmy et al., 2023; Naguib et al., 2024). Developing an effective vaccine against *Campylobacter* requires the identification of highly conserved immunogenic proteins capable of inducting robust, cross-protective immunity against different strains of *C. jejuni*. However, to date, no vaccine has been shown to offer complete protection against *Campylobacter* in chickens. Our group and others have demonstrated that subcutaneous administration of a multi-antigen vaccine, consisting of a crude mixture of *C. jejuni* outer membrane proteins (OMPs), induced systemic antibody response associated with a significant reduction in *Campylobacter* colonization (Pumtang-On et al., 2021; Naguib et al., 2024). This effect was further enhanced by concurrent administration of cytosine phosphorothioate guanine oligodeoxynucleotides (CpG ODN), an avian Toll-like receptor (TLR) 21 ligand. While the subcutaneous route is not considered feasible for mass administration in broiler production, it may be a practical option for breeders, given their relatively small population.

Expanding on these outcomes, this study aimed to investigate whether vaccinating layer breeders with a combination of *C. jejuni* OMPs and CpG ODN would exhibit the same efficacy in reducing *Campylobacter* shedding as observed for broilers and, more importantly, whether it could enhance and sustain high levels of *C. jejuni*-specific MDA in their offspring.

## Material and methods

### *Egg Incubation and Chicken Housing*

Forty 100-week-old white leghorn *Campylobacter*-seropositive layers and four roasters were housed at the Morgan Poultry Center at Clemson University. These layers are typically used for table egg production; however, in this study, they were utilized as breeders and are therefore referred to as breeders. Fertilized eggs were collected during the peak of IgY antibody levels and incubated in a sanitized egg incubator until hatching. The hatched chicks were transferred to the Godley-Snell Facility at Clemson University, where they were provided antibiotic- and additive-free diets *ad libitum*. All experimental procedures were approved by Clemson University's Institutional Animal Care and Use Committee (IACUC AUP 2022-0411)

### *Bacterial Strain and Culture Condition*

*C. jejuni* strain 81-176 was cultured as previously described (Taha-Abdelaziz et al., 2018a) with a minor modification. A loopful of glycerol frozen *C. jejuni* was briefly streaked onto Brain Heart Infusion (BHI) agar supplemented with Preston *Campylobacter* Selective Supplement (Oxoid, Basingstoke, Hampshire, UK). Afterward, the streaked plates were incubated in microaerophilic conditions (10% CO2, 5% O2, 85% N2) for 48 h at 37°C. Subsequently, several colonies were transferred to 5 mL of fresh BHI broth and incubated under the same conditions at 37°C for 48 h. Subsequently, a 100 mL fresh BHI broth was then inoculated with 1 mL of this culture. Following incubation under microaerophilic conditions, the bacterial suspension was centrifuged at 3,500 × g for 10 min, and then the obtained pellet was resuspended in phosphate-buffered saline (PBS, pH 7.4).

### ***Vaccine****P****reparation***

#### Preparation of Campylobacter OMPs

*C. jejuni* strain 81-176 OMPs were extracted using a previously published method (McCoy et al., 1975). In brief, three liters of *C. jejuni* suspension were prepared as described above, followed by centrifugation at 3,500 × g for 10 min and washing the bacterial cells with distilled water. Afterward, two grams of packed cells were suspended in 50 ml of 0.2 M glycine-hydrochloride (pH 2.2) and stirred at room temperature for 15 min. After centrifugation at 11,000 × g, the supernatant was collected, neutralized, and subjected to overnight dialysis against deionized water at 4°C. The protein concentration of the extracted OMPs was determined using a BCA Protein Assay Kit, and the samples were stored at -80°C for future use. Separation of proteins was confirmed with SDS-PAGE followed by Coomassie Blue staining.

#### CpG ODN

A synthetic class B 2007 CpG oligodeoxynucleotide (ODN) with a phosphorothioate backbone was purchased from Invivogen (San Diego, CA, USA). The CpG ODN was reconstituted in endotoxin-free water and then diluted to the required concentrations (50 µg /dose) using PBS.

### ***ExperimentalDesign***

#### Breeder *F*lock (Parents)

**Fig. 1:**
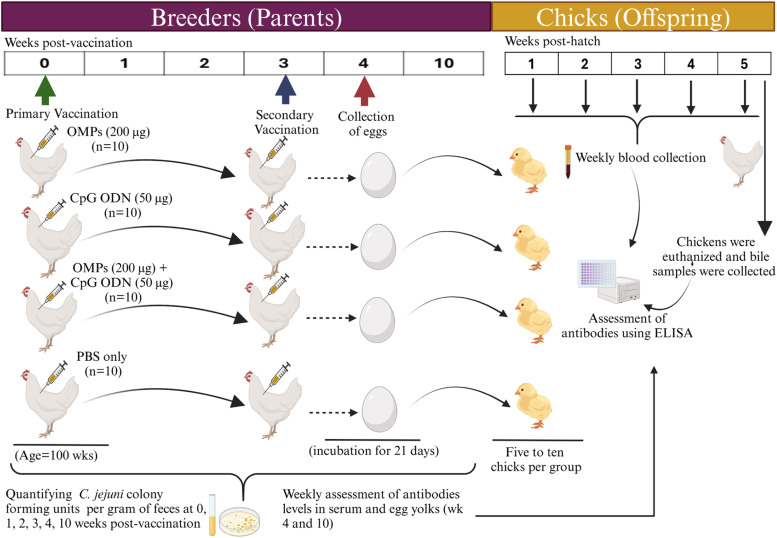
Illustration of the experimental design. Forty 100-week-old layer breeders were randomly divided into four groups (G1-G4) of ten breeders each, with one rooster per group. Breeders were vaccinated subcutaneously with either 200 μg *C. jejuni* OMPs, 50 μg CpG ODN, their combination, or PBS (serve as negative control). Three weeks later, a booster dose of the corresponding vaccine was injected SC. Weekly blood and fecal samples were collected. Eggs were collected at four weeks post-primary vaccination and incubated in an egg incubator until hatching. Hatched chicks were assigned into different groups according to their parents’ vaccination (5-10 chicks per group). Blood samples were collected weekly until week five of age. At day 35 of age, all chicks were euthanized, and bile samples were collected.

Forty-four 100-week-old white leghorns (40 breeders and four roosters) were distributed into four pens, with ten breeders and a rooster per pen. All groups were injected subcutaneously (SC) with different vaccine formulations, including 200 μg *C. jejuni* OMPs and 50 μg CpG-ODN, individually or their combination, or PBS as negative control, as depicted in Table 1. A booster dose of the corresponding vaccine was injected SC three weeks after primary vaccination. Blood samples and fresh feces were collected before vaccination and at 1, 2, 3, 4, and 10 weeks post-primary vaccination. *C. jejuni*-specific IgY levels were measured in the breeders' sera to determine the optimal time (peak of IgY level) for egg collection for subsequent procedures. Eggs were collected from each group at four weeks post-primary vaccination (one week post-secondary vaccination), labeled and transferred to a sterilized incubator until embryonic day 18, and then moved to a sterilized hatchery until hatching. The detailed experimental procedure is illustrated in Fig. 1.

**Table 1:** Experimental design for breeders.

Group	Treatment	Breeders (n)/group	Primary vaccination			Secondary vaccination
			Age (weeks)	Route	Volume (ml)	Route/volume (ml)/age (wk)
1	200 µg *C. jejuni* OMPs	10	100	SC	0.5	SC/0.5/103
2	50 µg CpG-ODN	10	100	SC	0.5	SC/0.5/103
3	200 µg *C. jejuni* OMPs + 50 µg CpG-ODN	10	100	SC	0.5	SC/0.5/103
4	PBS	10	100	SC	0.5	SC/0.5/103

#### Layer Chicks (Offspring)

After hatching, all chicks were moved to the Godley-Snell facility at Clemson University. Due to the relatively low hatchability rate, five to ten chicks per group were housed in separate kennels according to their parents’ vaccination. Chicks were fed antibiotic- and additive-free diets *ad libitum*. All chicks were bled weekly, starting from the first week post-hatch until the fifth week of age. At the end of the experiment (day 35 of age), all chickens were euthanized, and bile contents were collected.

#### Enzyme-Linked Immunosorbent Assay (ELISA) for *M*easuring Campylobacter-specific Antibody Levels in Breeders’ Aera, Egg Yolks and Hatched Chicks

IgY, IgM and IgA antibody levels were measured as previously described (Hodgins et al., 2015). Briefly, Maxisorp 96-well plates (Thermo Fisher Scientific, Rochester, NY, USA) were coated with OMPs of *C. jejuni* (0.39 µg/100 µL) in PBS (pH 7.4) and incubated at 37°C for two hours. After washing four times with PBS containing 0.05% Tween 20, the plates were blocked with blocking buffer containing 0.5% pig gelatin (Sigma, St. Louis, MO, USA) and 0.05% Tween 20 in PBS and incubated at 37°C for one hour. Afterward, breeders' sera were diluted 1/1000 while offspring' sera were diluted 1/10 in a dilution buffer (PBS containing 1.5% Tween-20 and 0.29 M NaCl) and 100 µL was added to wells in duplicate; then the plates were incubated for one hour at 37°C. After four times washing, 100 µL of goat-anti-chicken HRP-conjugated IgY (Sigma, USA) or IgA (Invitrogen, USA) or IgM (Invitrogen, USA) antibodies were added at a dilution of 1/4000, 1/4000 or 1/10,000, respectively, and then the plates were incubated at 37°C for 30 min. After washing the plates four times with the washing buffer, 100 µL of ABTS (2, 2′-azino-di (3-ethyl-benzthiazoline-6-sulfonate)) substrate (Life Technologies, Frederick, MD, USA) was added to each well. After 30 min of incubation at room temperature, the reaction was stopped by adding 1% sodium dodecyl sulfate (Bio-Rad, Hercules, CA, USA) to wells. The optical densities (OD) were measured at 405 nm.

#### Campylobacter-specific IgY, IgA, and IgM Antibody Levels in Egg Yolks

*C. jejuni*-specific antibody levels in egg yolks were measured, as previously described (Hermans et al., 2014), with minor modifications. Egg yolks were diluted 1/5 (vol/vol) in HBSS. After thoroughly mixing, the suspension was incubated at 4°C overnight. The water-soluble supernatant was then collected and diluted 1/1000 in a dilution buffer. Antibody IgY, IgM, and IgA levels were quantified using ELISA, as described above.

#### Quantification of C. jejuni Colony Count in Fecal Droppings

To quantify the *C. jejuni* count in vaccinated and non-vaccinated breeders, fresh fecal samples were collected from eight breeders before vaccination to determine the colonization rate of *Campylobacter* and at 1, 2, 3, 4, and 10 weeks post-primary vaccination. Afterward, ten-fold serial dilutions were performed for each sample in PBS, and each dilution was plated on BHI agar containing Preston *Campylobacter* selective supplement. The plates were then incubated in microaerophilic conditions (85% N2, 10% CO2, and 5% O2) for 48 h at 37°C. Following incubation, the fecal *C. jejuni* colony-forming units (CFUs) were quantified and calculated as log_10_ *C. jejuni* per gram of feces as previously described (Taha-Abdelaziz et al., 2018a).

#### Assessing the Vertical Transmission of C. jejuni in Eggs

To determine the possibility of the vertical transmission of *C. jejuni* from breeders to their eggs, ten eggs per group were collected at four weeks post-primary vaccination. Afterward, eggshells were surface sterilized using 70% ethanol to eliminate external contamination; then 100 µL of egg yolk and 100 µL egg white were individually streaked onto BHI agar supplemented with Preston *Campylobacter* selective supplement. The agar plates were then incubated under the same conditions mentioned above. Following 48 h of incubation, all plates were then examined for bacterial growth.

#### Statistical Analysis

After analyzing the data using JMP® Pro 17.1.0, graphs were generated using GraphPad Prism. V5.0. The Shapiro–Wilk test was used to check the normality of the CFUs and antibody levels data. Parametric and nonparametric tests were employed to evaluate the vaccination effects on colony count and antibody. The normally distributed data were analyzed using One-way ANOVA followed by Tukey's post hoc test. Non-normally distributed data were analyzed using Kruskal–Wallis testing followed by Dunn's test. The colony count or antibody levels were presented as the mean value ± standard error of the mean (SEM). P values less than 0.05 (*P < 0.05*) are considered significant for all tests.

## Results

### *The Effect of the SC Vaccination of Breeders with Various Vaccine Formulations on C. jejuni Shedding*

**Fig. 2:**
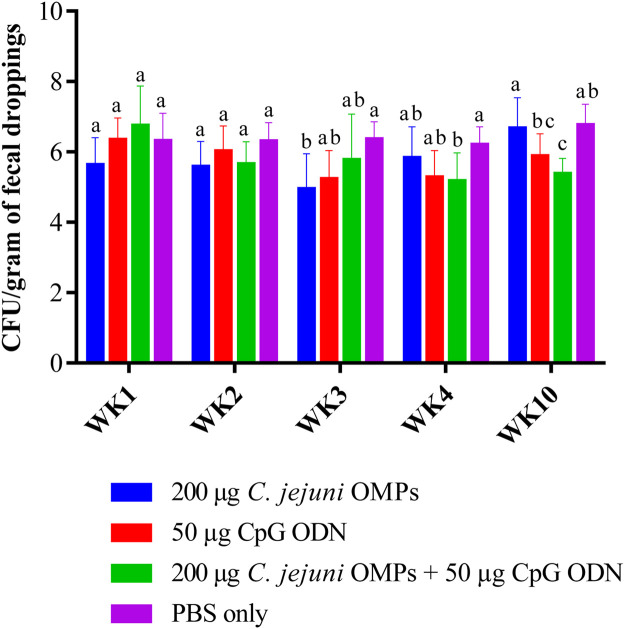
*C. jejuni* colony count per gram of feces. Fresh fecal droppings were collected at 1, 2, 3, 4, and 10 weeks post-initial SC vaccination. Bars with the same letters (a-c) show no significant differences between groups, whereas those with different letters indicate significant differences (*P < 0.05*). OMPs = outer membrane proteins. CpG ODN = synthetic single-stranded oligodeoxynucleotides (ODNs) containing unmethylated CpG motifs.

*Campylobacter* was detected at a level of 6.7 ± 0.4 log_10_ in breeders prior to vaccination (Table 1). No significant reduction in *Campylobacter* counts was observed among the vaccinated and non-vaccinated groups during the first two weeks post-primary vaccination. However, a significant reduction in *Campylobacter* counts was observed in the following weeks in the groups that were vaccinated with *C. jejuni* OMPs alone or in combination with CpG ODN *(P < 0.05*) (Fig. 2). SC vaccination of breeders with the combination of *C. jejuni* OMPs and CpG-ODN significantly reduced *C. jejuni* shedding by 1.02 and 1.37 log_10_ (*P < 0.05*) at weeks four and ten post-primary vaccination, respectively. Vaccination with the *C. jejuni* OMPs alone significantly reduced *C. jejuni* shedding by 1.4 log_10_ (*P < 0.03*) at three weeks post-vaccination; however, no further reduction was observed in the following weeks. On the other hand, no significant reduction in *C. jejuni* counts was observed in the group receiving CpG-ODN alone at any of the measured time points (Table 2).

**Table 2:** *Campylobacter* counts in fecal droppings.

Group	Colony count (log_10_)					
	Before vaccination	Post-primary vaccination				
	WK0	WK1	WK2	WK3	WK4	WK10
	6.7±0.4	-	-	-	-	-
200 µg OMP	-	5.6±0.3	5.6±0.2	5±0.4*	5.8±0.3	6.7±0.3
200 µg CpG ODN	-	6.4±0.2	6±0.3	5.2±0.3	5.3±0.3	5.9±0.2
200 µg OMP + 50 µg ODN	-	6.8±0.4	5.7±0.2	5.8±0.5	5.2±0.3*	5.4±0.2*
PBS (control)	-	6.3±0.3	6.3±0.2	6.4±0.2	6.2±0.2	6.8±0.2

⁎ Indicate a significant difference between the vaccinated and unvaccinated control group (P < 0.05).

### *Vertical Transmission of C. jejnui from Breeders to Eggs*

No *C. jejuni* colonies were detected in any sample in all vaccinated and non-vaccinated groups.

### *The Effect of the SC Vaccination of Breeder with Different Vaccine Formulations on the Serum Antibody Levels*

#### Campylobacter-specific IgY Antibody Levels in Breeders’ Sera

**Fig. 3:**
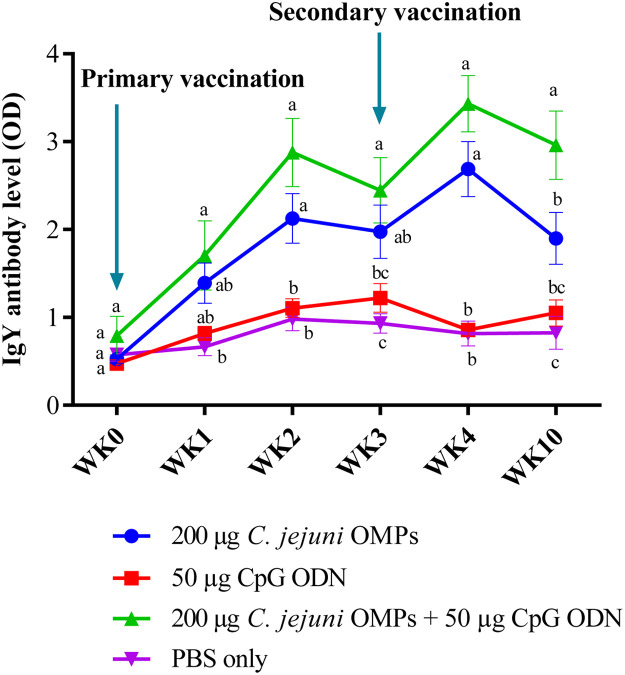
*Campylobacter*-specific IgY antibody levels in sera of vaccinated breeders. Breeders were vaccinated SC with 200 µg *C. jejuni* OMPs or 50 µg CpG ODN or their combination or PBS. Booster doses were administered three weeks post-primary vaccination. Blood samples were collected pre-vaccination (week 0), weekly for four weeks post-primary vaccination, and at week ten. Bars represent mean optical density (OD) ± standard error at 405 nm for ten birds per group. Bars with the same letters (a-c) show no significant differences between groups, whereas those with different letters indicate significant differences (*P < 0.05*). OMPs = outer membrane proteins. CpG ODN = synthetic single-stranded oligodeoxynucleotides (ODNs) containing unmethylated CpG motifs.

No significant differences in IgY levels were observed among the layer breeders before primary vaccination. Breeders vaccinated with the combination of *C. jejuni* OMPs and CpG ODN exhibited higher IgY antibody levels after one week of post-primary vaccination (*P < 0.01*). A further increase in the IgY levels was observed after the second week (*P < 0.0001*), followed by a slight decline by the third week, but remained significantly higher than the non-vaccinated group (*P < 0.0001*). Following the administration of the booster dose, IgY levels significantly increased (*P < 0.0001*) during the first week after the secondary vaccination (the fourth week after primary vaccination) and then slightly declined by week ten post-primary vaccination but remained significantly higher than those of the non-vaccinated group (*P < 0.0001*) (Fig. 3).

A similar, but lower, trend was observed for breeders vaccinated with *C. jejuni* OMPs alone. While no significant differences were observed in the IgY levels between the vaccinated and non-vaccinated breeders at week one of primary vaccination, significantly higher levels (*P < 0.015*) were observed in the following weeks, including weeks three (*P < 0.03*), four (*P < 0.0001*), and ten (*P < 0.04*) post-vaccinations. IgY levels in the group receiving the combination of *C. jejuni* OMPs and CpG-ODN were significantly higher (*P < 0.03*) than the group receiving OMPs alone at ten weeks post-primary vaccination. No significant differences in the IgY levels were observed between the group that received CpG ODN alone and the non-vaccinated group at any of the measured time points.

#### Campylobacter-Specific IgM Antibody Levels in Breeders’ Sera

**Fig. 4:**
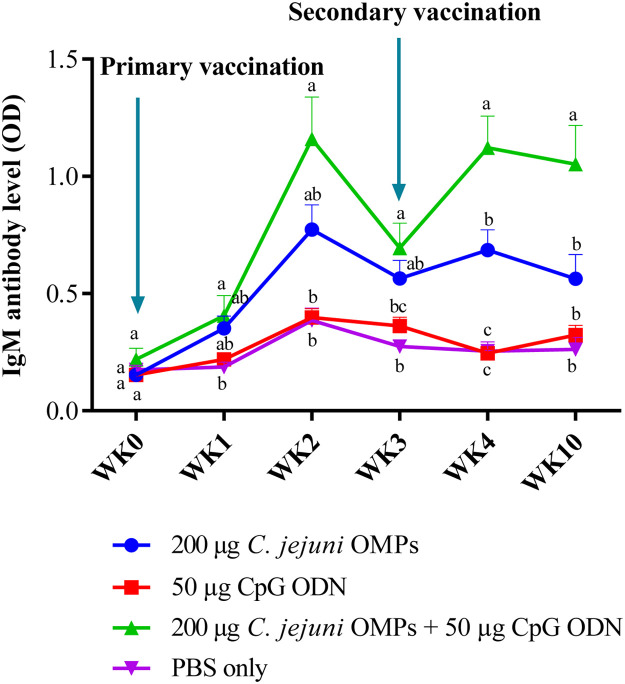
*Campylobacter*-specific IgM antibody levels in sera vaccinated hens. Breeders were vaccinated SC with 200 µg *C. jejuni* OMPs or 50 µg CpG ODN or their combination or PBS. Booster doses were administered three weeks post-primary vaccination. Blood samples were collected pre-vaccination (week 0), weekly for four weeks post-primary vaccination, and at week ten. Bars represent mean optical density (OD) ± standard error at 405 nm for ten birds per group. Bars with the same letters (a-c) show no significant differences between groups, whereas those with different letters indicate significant differences (*P < 0.05*). OMPs = outer membrane proteins. CpG ODN = synthetic single-stranded oligodeoxynucleotides (ODNs) containing unmethylated CpG motifs.

IgM levels exhibited a similar trend to that of IgY antibody. No significant differences in the IgY levels were observed among the layer breeders before primary vaccination. Vaccination of breeders with the combination of *C. jejuni* OMPs and CpG ODN induced significantly higher IgM levels after the first week of primary vaccination (*P < 0.02*), which peaked in the second week (*P < 0.0001*) and slightly declined but remained significantly higher than the non-vaccinated group by the third week of primary vaccination (*P < 0.007*). One week after the secondary vaccination (the fourth week after primary vaccination), IgM levels surged significantly (*P < 0.0001*) and then slightly declined by week ten post-primary vaccination (seventh week post-secondary vaccination), reaching levels similar to those observed at week three post-primary vaccination (*P < 0.0001*) (Fig. 4). The combination of *C. jejuni* OMPs and CpG ODN significantly elevated the IgM levels more than the OMPs alone at weeks four (*P < 0.004*) and ten (*P < 0.01*) post-primary vaccination. Serum IgM levels in the breeders receiving OMPs alone were significantly higher (*P < 0.004*) at week four post-primary vaccination than in the control group, with no significant differences observed in the preceding or subsequent weeks. No significant differences in IgM levels were observed between the group that received CpG ODN alone and the non-vaccinated group at any of the measured time points.

#### Campylobacter-Specific IgA Antibody Levels in Breeders’ Sera

**Fig. 5:**
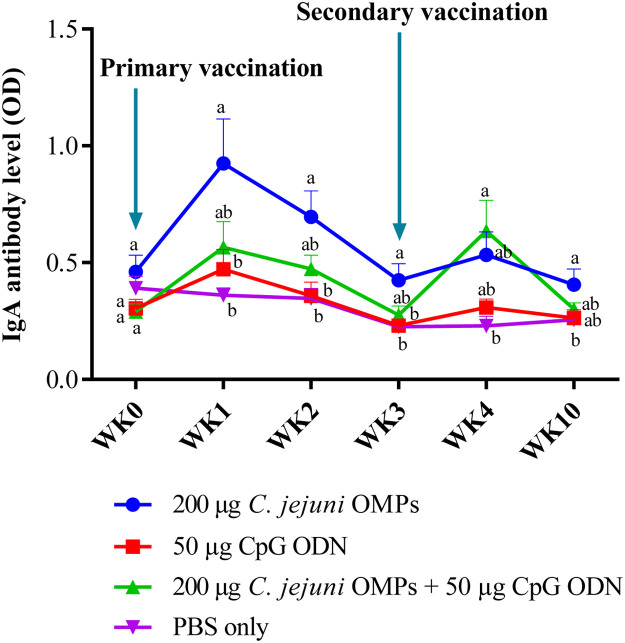
*Campylobacter*-specific IgA antibody levels in sera of vaccinated breeders. Breeders were vaccinated SC with 200 µg *C. jejuni* OMPs or 50 µg CpG ODN or their combination or PBS. Booster doses were administered three weeks post-primary vaccination. Blood samples were collected pre-vaccination (week 0), weekly for four weeks post-primary vaccination, and at week ten. Bars represent mean optical density (OD) ± standard error at 405 nm for ten birds per group. Bars with the same letters show no significant differences between groups, whereas those with different letters indicate significant differences (*P < 0.05*). OMPs = outer membrane proteins. CpG ODN = synthetic single-stranded oligodeoxynucleotides (ODNs) containing unmethylated CpG motifs.

No significant differences in the IgY levels were observed among the layer breeders before primary vaccination. Significantly elevated IgA levels (*P < 0.009*) were observed in the group vaccinated with *C. jejuni* OMPs alone in the first week following primary vaccination. This group maintained higher IgA levels in the second (*P < 0.008*), third weeks (*P < 0.01*) and tenth week (*P < 0.04*) after primary vaccination compared to the control group (Fig. 5). The group vaccinated with the combination of *C. jejuni* OMPs and CpG ODN had higher IgA levels only at week four post-primary vaccination (*P < 0.01*). Similar to IgY and IgM, no significant differences in IgA levels were observed between the group that received CpG ODN alone and the non-vaccinated group at any of the measured time points.

### The Effect of the SC Vaccination of Breeders with Various Vaccine Formulations on their Egg Yolk Antibody Levels

#### Campylobacter-specific IgY Antibody Levels in Egg Yolk

**Fig. 6:**
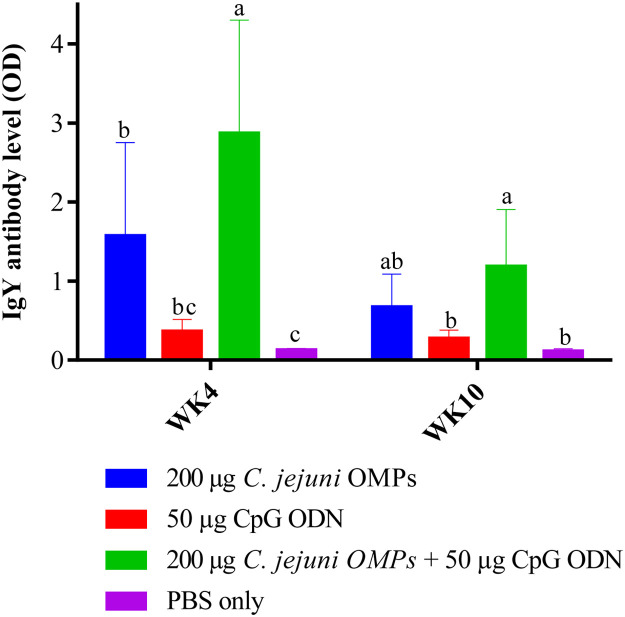
*Campylobacter*-specific IgY antibody levels in egg yolks from vaccinated breeders. Breeders were vaccinated SC with 200 µg *C. jejuni* OMPs or 50 µg CpG ODN or their combination or PBS. Booster doses were administered three weeks post-primary vaccination. Eggs were collected at four and ten weeks post-primary vaccination. The aqueous yolk portion was separated, and IgY antibody levels were measured using ELISA. Bars represent mean optical density (OD) ± standard error at 405 nm for ten birds per group. Bars with the same letters (a-c) show no significant differences between groups, whereas those with different letters indicate significant differences (*P < 0.05*). OMPs = outer membrane proteins. CpG ODN = synthetic single-stranded oligodeoxynucleotides (ODNs) containing unmethylated CpG motifs.

*C. jejuni*-specific IgY levels in egg yolk differed significantly among vaccinated and non-vaccinated groups. Egg yolks from breeders vaccinated with *C. jejuni* OMPs alone or the combination of *C. jejuni* OMPs and CpG ODN had significantly higher *C. jejuni*-specific IgY levels compared to the control group at weeks four (*P < 0.02*) and ten (*P < 0.0001*) post-primary vaccination (Fig. 6). However, egg yolks from the breeders that received the combination of *C. jejuni* OMPs and CpG ODN maintained elevated IgY levels until week ten post-primary vaccination *(P < 0.0001*). No significant differences in the egg yolk IgY levels were observed in the group that received CpG ODN alone compared to the non-vaccinated group at weeks four and ten post-primary vaccination.

#### Campylobacter-specific IgM Antibody Levels in Egg Yolk

**Fig. 7:**
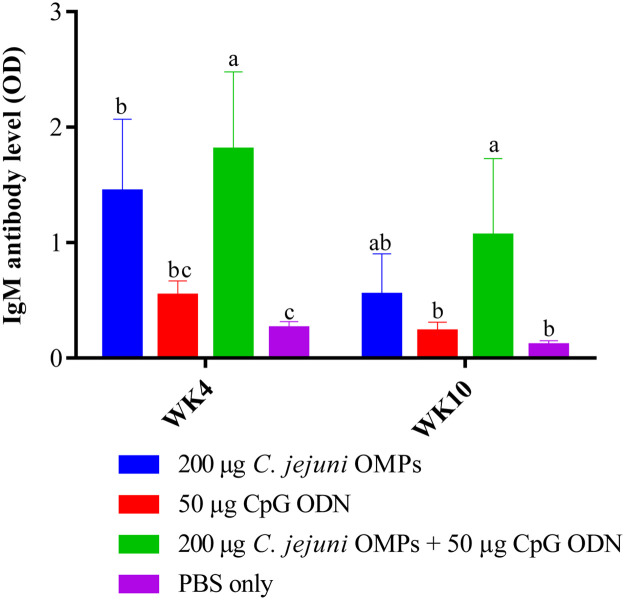
*Campylobacter*-specific IgM antibody levels in egg yolks from vaccinated breeders. Breeders were vaccinated SC with 200 µg *C. jejuni* OMPs or 50 µg CpG ODN or their combination or PBS. Booster doses were administered three weeks post-primary vaccination. Eggs were collected at four and ten weeks post-primary vaccination. The aqueous portion of the yolk was separated, and IgM antibody levels were measured using ELISA. Bars represent mean optical density (OD) ± standard error at 405 nm for ten birds per group. Bars with the same letters (a-c) show no significant differences between groups, whereas those with different letters indicate significant differences (*P < 0.05*). OMPs = outer membrane proteins. CpG ODN = synthetic single-stranded oligodeoxynucleotides (ODNs) containing unmethylated CpG motifs.

In a similar pattern to IgY levels, IgM levels varied significantly among the vaccinated and non-vaccinated groups. Administering the combination of *C. jejuni* OMPs and CpG ODN or OMPs alone significantly increased *C. jejuni*-specific IgM levels in egg yolks at week four (*P < 0.0001*) and ten post-primary vaccination (*P < 0.0001*) (Fig. 7), with egg yolks from the combined group of breeders maintained elevated IgM levels until week ten post-primary vaccination (*P < 0.0001*). No significant differences in the IgM levels were observed in the group that received CpG ODN alone compared to the non-vaccinated group, at weeks four and ten post-primary vaccination.

#### Campylobacter-specific IgA Antibody Levels in Egg Yolk

No *C. jejuni*-specific IgA antibodies were detected in egg yolks of all groups at all time points.

### The effect of the SC Vaccination of Breeders with Various Vaccine Formulations on MDA Levels in Their Offspring (Hatched Chicks)

#### Campylobacter-specific IgY MDA Levels in Hatched Chicks’ Sera

**Fig. 8:**
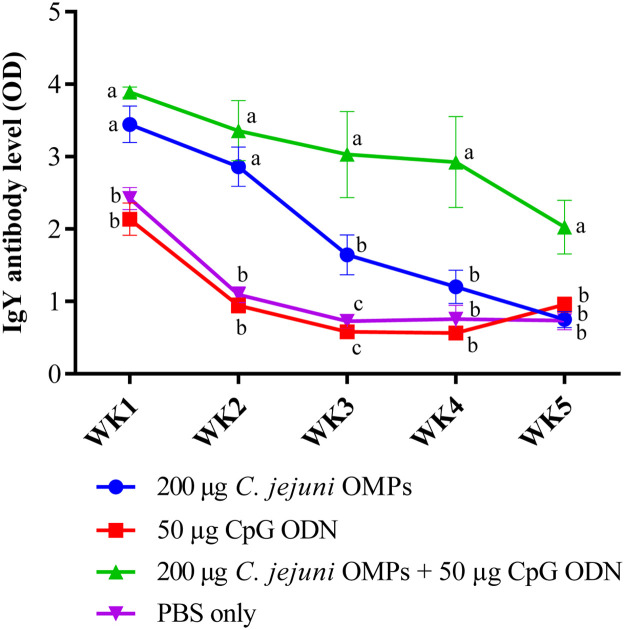
*Campylobacter*-specific IgY maternally derived antibody levels in sera of offspring of vaccinated breeders. Hatched layer chicks were housed in four separate pens according to their parental vaccination. Blood samples were collected weekly, from the first week post-hatch until the end of the experiment at day 35 of age. Sera were subsequently separated, and IgY antibody levels were measured using ELISA. Bars represent mean optical density (OD) ± standard error at 405 nm for 5-10 birds per group. Bars with the same letters (a-c) show no significant differences between groups, whereas those with different letters indicate significant differences (*P < 0.05*). OMPs = outer membrane proteins. CpG ODN = synthetic single-stranded oligodeoxynucleotides (ODNs) containing unmethylated CpG motifs.

*C. jejuni*-specific IgY MDA levels differed significantly between chicks from vaccinated and non-vaccinated breeders. After the first week post-hatch, significantly elevated IgY levels were detected in the sera of chicks from the parent group vaccinated with the combination of *C. jejuni* OMPs and CpG ODN (*P < 0.001*) (Fig. 8). Notably, this group showed a slight weekly decrease in IgY levels but remained significantly higher compared to the control group throughout the entire 35-day experimental period. The differences remained statistically significant at weeks two (*P < 0.0001*), three (*P < 0.0001*), four (*P < 0.0001*), and five (*P <0.001*) post-hatch. Chicks from the group vaccinated with *C. jejuni* OMPs alone had significantly higher MDA IgY levels at the first week post-hatch (*P < 0.005*), which declined by second (*P < 0.0001*) and third (*P < 0.03*) weeks post-hatch but remained significantly higher than those from the non-vaccinated group. No significant differences were observed in the IgY levels between chickens from the group receiving CpG ODN alone and the non-vaccinated group.

#### Campylobacter-specific IgM MDA Levels in Hatched Chicks’ Sera

**Fig. 9:**
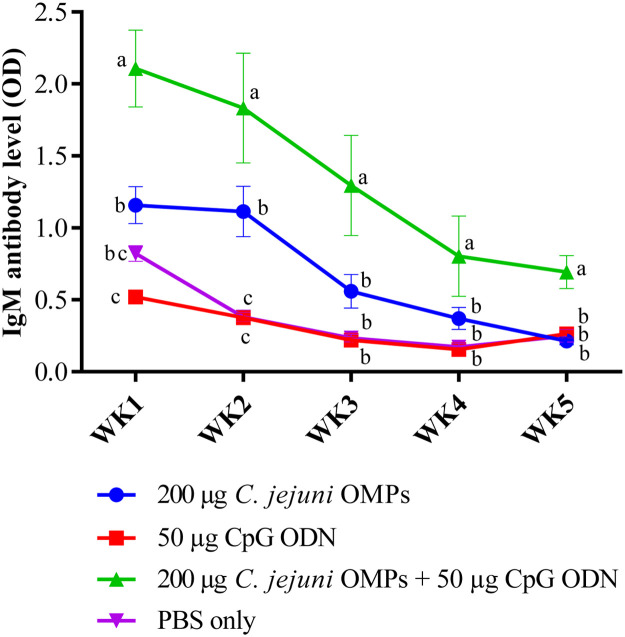
*Campylobacter*-specific IgM maternally derived antibody levels in sera of offspring of vaccinated breeders. Hatched chicks were housed in four separate pens according to their parental vaccination. Blood samples were collected weekly, from the first week post-hatch until the end of the experiment at 35 days old. Sera were subsequently separated, and IgM antibody levels were measured using ELISA. Bars represent mean optical density (OD) ± standard error at 405 nm for 5-10 birds per group Bars with the same letters (a-c) show no significant differences between groups, whereas those with different letters indicate significant differences (*P < 0.05*). OMPs = outer membrane proteins. CpG ODN = synthetic single-stranded oligodeoxynucleotides (ODNs) containing unmethylated CpG motifs.

A similar trend in IgM levels was observed as for IgY; *C. jejuni*-specific IgM levels were higher in the chicks from the group receiving the combination of *C. jejuni* OMPs and CpG ODN in the first week post-hatch. These levels then slightly decreased weekly but remained significantly higher than those in the non-vaccinated group throughout the entire 35-day experimental period: week one (*P < 0.0001*), week two (*P < 0.0001*), week three (*P < 0.0001*), week four (*P < 0.0007*), and week five (*P < 0.0001*). (Fig. 9). On the other hand, chicks from the *C. jejuni* OMPs-vaccinated group showed higher IgM levels (*P < 0.003*) only in the second week post-hatch. No significant differences were observed in the IgM levels between chickens from the group receiving CpG ODN alone and the non-vaccinated group.

#### Campylobacter-specific IgA Levels in the Bile of Hatched Chicks

**Fig. 10:**
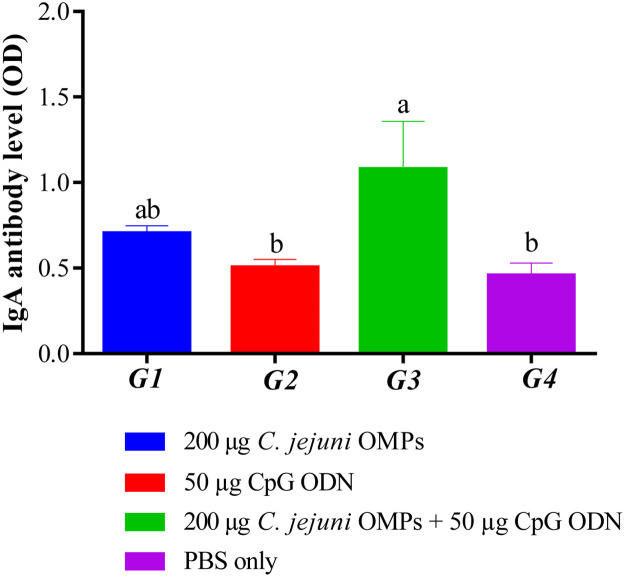
*Campylobacter*-specific IgA antibody levels in the bile of offspring from vaccinated breeders. Hatched chicks were housed in four separate pens according to their parental vaccination. All chickens were euthanized at day 35 of age. Bile samples were collected, and IgA antibody levels were measured using ELISA. Bars represent mean optical density (OD) ± standard error at 405 nm for 5-10 birds per group. Bars with the same letters show no significant differences between groups, whereas those with different letters indicate significant differences (*P < 0.05*). OMPs = outer membrane proteins. CpG ODN = synthetic single-stranded oligodeoxynucleotides (ODNs) containing unmethylated CpG motifs.

Significantly higher IgA levels in bile were observed in chickens from the breeders vaccinated with the combination of *C. jejuni* OMPs and CpG ODN (*P < 0.0001*) (Fig. 10) at day 35 of age. No significant differences in IgA levels were observed in the biles among the chickens from the other vaccinated and non-vaccinated groups.

## Discussion

The limited immune capabilities of hatched chicks, particularly during the first two weeks of their life, make them highly vulnerable to infection by bacterial and viral pathogens (Taha-Abdelaziz et al., 2018b). Providing passive immunity during this critical period, either in the form of MDA from vaccinated mothers or through the administration of hyperimmune eggs, has been shown to provide protection against a wide range of pathogens (Eterradossi et al., 1997; Kariyawasam et al., 2004; Chalghoumi et al., 2009). In the context of *Campylobacter*, previous studies suggest dietary inclusion of hyperimmune egg yolk from hens vaccinated with *Campylobacter* whole cell lysate or its hydrophobic protein fraction or bacterin subunit vaccine to reduce *Campylobacter* colonization (Vandeputte et al., 2019; Vandeputte et al., 2020)*.* However, many factors limit the application of this approach in poultry production, including (I) the instability of antibodies inside the gut of chickens due to the negative effect of proteases and extreme pH conditions (Shimizu et al., 1992; Wang et al., 2021a; Wang et al., 2021b), (II) the absorption of antibodies from the intestine rapidly diminishes as chickens age (Yokoyama et al., 1993; Wickramasuriya et al., 2022), and (III) the need for large quantities of eggs limits the ability to scale up this approach.

While the role of MDA in protecting chicks during the first two to three weeks of their life against *Campylobacter* infection is well-documented, research on boosting their levels and ensuring long-term effectiveness remains limited. A recent study by Haems and colleagues demonstrated that broiler breeders required multiple doses of a bacterin or subunit *Campylobacter* vaccine to sustain high levels of antibodies in their sera and egg yolk (Haems et al., 2024). While these vaccination strategies successfully enhanced antibody production in breeders, they showed limited efficacy in enhancing the resistance of their chicks to *Campylobacter* infection. This was attributed to the inadequate levels of MDA, which did not persist for an extended period. Thus, it is essential not only to elevate antibody levels in the breeders' sera but also to ensure that their offspring maintain high MDA levels for extended periods to ensure prolonged protection.

This study evaluated the potential of a subunit multi-antigen vaccine, comprising *C. jejuni* OMPs and CpG ODN as an adjuvant, to reduce *Campylobacter* shedding and enhance *C. jejuni*-specific antibody levels in chicken breeders and their offspring over a five week period. It is worth noting that the layer breeders used in this study were naturally colonized with *Campylobacter* and exhibited high serum levels of *Campylobacter*-specific antibodies. Vaccinating these breeders with either *C. jejuni* OMPs alone or in combination with CpG ODN significantly increased sera IgY and IgM antibody levels and reduced *Campylobacter* shedding by over 1 log_10__._ While no substantial differences in *Campylobacter* shedding were observed between these groups, the combination of *C. jejuni* OMPs and CpG ODN appeared to elicit relatively higher levels of these antibodies compared to *C. jejuni* OMPs alone. Indeed, numerous *in vitro* and *in vivo* studies indicated that CpG-ODN is a potent immunostimulator and highlighted its potential as a vaccine adjuvant or as a stand-alone antimicrobial agent (Gomis et al., 2003; Brownlie et al., 2009; Keestra et al., 2010; Gunawardana et al., 2015; Taha-Abdelaziz et al., 2018c). For instance, administering CpG-ODN through different routes, including intrapulmonary, intramuscular and *in ovo* routes, has been shown to enhance chickens' resistance to *E. coli, Clostridium perfringens* and *Salmonella* Typhimurium infections (Gunawardana et al., 2020; Goonewardene et al., 2021; Gautam et al., 2024; Subhasinghe et al., 2024). Consistent with these observations, our group has previously demonstrated the potential of nanoparticle-encapsulated CpG ODN in eliciting robust immune responses in the cecal tonsils and ileum and significantly reducing *Campylobacter* when orally delivered to broiler chickens (Taha-Abdelaziz et al., 2017). Moreover, in a most recent study, concurrent subcutaneous administration of *C. jejuni* OMPs and CpG ODN to broiler chickens has been shown to induce the expression of interferon (IFN)-γ, interleukin (IL)-1β, and IL-13 in the spleen, significantly elevated serum IgM and IgY antibody levels, and reduced cecal *C. jejuni* counts by approximately 1.2 log_10_ (Naguib et al., 2024). While the specific immunogenic protein in the OMPs responsible for the observed effects remains unclear, the results of this study confirm and extend our recent findings, highlighting the adjuvant potential of CpG ODN in enhancing the immunogenicity and protective efficacy of *C. jejuni* OMPs.

In addition to exploring the potential of these vaccines in minimizing the horizontal transmission of *Campylobacter* by reducing its shedding in breeders, we also aimed to investigate whether they could control the vertical transmission via eggs. Indeed, the vertical transmission of *C. jejuni* in poultry remains debatable. In contrast to previous studies suggesting the possibility of vertical transmission from breeder flocks to their progeny (Sahin et al., 2002b; Cox et al., 2012), our results revealed that despite the high colonization rates in breeders used in this study, no bacterial colonies were detected in the egg yolks or whites of the tested samples. However, these results should be interpreted with caution, as traditional culture techniques may lack the sensitivity needed to detect low levels of *Campylobacter* contamination. Nonetheless, these findings align with those of Callicott et al. (2006), who used molecular methods to examine approximately 60,000 progeny parent breeders hatched from eggs of *Campylobacter*-positive parent flocks and found no indication of vertical transmission. Collectively, these data support the notion that vertical transmission does not occur from infected parents to their offspring.

The significant elevation of antibody levels in the vaccinated breeders prompted further investigation into whether their offspring would sustain elevated levels of these antibodies in their sera over an extended period. Interestingly, similar patterns for the antibody levels were observed in hatched chicks from the vaccinated breeders, with the chicks belonging to the group vaccinated with the combination of *C. jejuni* OMPs and CpG ODN maintained significantly higher levels in their sera for up to five weeks post-hatch. Although IgA antibody was undetectable in egg yolks from both vaccinated and non-vaccinated groups, likely due to technical issues, chicks from breeders vaccinated with the combination of *C. jejuni* OMPs and CpG ODN showed higher IgA levels in their biles.

Despite their role in providing crucial protection to offspring against infection during their early life, the short-lived passive immunity conferred by transferred MDA levels necessitates either boosting their levels in breeders for long-term protection or vaccinating the offspring directly. The waning of MDA in chicks from breeders vaccinated against various poultry pathogens using commercial vaccines was previously studied (Gharaibeh and Mahmoud, 2013). It has been reported that the half-life time for MDA in chick serum ranges from 3.8 to 7 days, and breeders' vaccination was found to extend their detection to 25 days. A recent study reported that MDA was detachable in three-week-old chicks from broiler breeders vaccinated with a *Campylobacter* subunit vaccine (Haems et al., 2023). In the present study, chicks from breeders vaccinated with the combination of *C. jejuni* OMPs and CpG ODN exhibited significantly elevated MDA levels compared to the control group, with these levels remaining significantly elevated until the end of the experiment at 35 days of age. However, whether these high antibody levels would effectively curb *Campylobacter* infection was not investigated in this study, as the unexpectedly low hatchability rate, likely due to the relatively older age of the breeders, limited further evaluation. Nonetheless, even with the small number of birds per group, significant statistical differences were observed. Furthermore, the observed persistence of high MDA levels until the fifth week of age, which were comparable to those of control birds at the first week of age, could serve as a critical milestone for further research on their protective efficacy against *C. jejuni* infection.

In conclusion, this proof-of-concept study confirms and expands upon our earlier findings supporting the vaccine formulation combining *C. jejuni* OMPs and CpG ODN as a promising strategy for reducing *Campylobacter* colonization in both broilers and breeders. More importantly, it provides valuable insights into its potential to enhance and sustain elevated levels of MDA in their offspring up to day 35 of age. However, further investigation is warranted to assess the protective efficacy of this maternally derived immunity to curb *Campylobacter* infection in commercial broilers.

## Disclosures

The authors declare that there are no conflicts of interest.
